# Chemoreactive Nanotherapeutics by Metal Peroxide Based Nanomedicine

**DOI:** 10.1002/advs.202000494

**Published:** 2020-12-03

**Authors:** Hui Hu, Luodan Yu, Xiaoqin Qian, Yu Chen, Baoding Chen, Yuehua Li

**Affiliations:** ^1^ Medmaterial Research Center Jiangsu University Affiliated People's Hospital Zhenjiang 212002 P. R. China; ^2^ Institute of Diagnostic and Interventional Radiology Shanghai Jiao Tong University Affiliated Sixth People's Hospital Shanghai 200233 P. R. China; ^3^ School of Life Sciences Shanghai University Shanghai 200444 P. R. China; ^4^ Department of Medical Ultrasound The Affiliated Hospital of Jiangsu University Zhenjiang 212001 P. R. China

**Keywords:** chemoreactive nanotherapeutics, H_2_O_2_, metal peroxides, O_2_, reactive nanomedicine

## Abstract

The advances of nanobiotechnology and nanomedicine enable the triggering of in situ chemical reactions in disease microenvironment for achieving disease‐specific nanotherapeutics with both intriguing therapeutic efficacy and mitigated side effects. Metal peroxide based nanoparticles, as one of the important but generally ignored categories of metal‐involved nanosystems, can function as the solid precursors to produce oxygen (O_2_) and hydrogen peroxide (H_2_O_2_) through simple chemical reactions, both of which are the important chemical species for enhancing the therapeutic outcome of versatile modalities, accompanied with the unique bioactivity of metal ion based components. This progress report summarizes and discusses the most representative paradigms of metal peroxides in chemoreactive nanomedicine, including copper peroxide (CuO_2_), calcium peroxide (CaO_2_), magnesium peroxide (MgO_2_), zinc peroxide (ZnO_2_), barium peroxide (BaO_2_), and titanium peroxide (TiO_x_) nanosystems. Their reactions and corresponding products have been broadly explored in versatile disease treatments, including catalytic nanotherapeutics, photodynamic therapy, radiation therapy, antibacterial infection, tissue regeneration, and some synergistically therapeutic applications. This progress report particularly focuses on the underlying reaction mechanisms on enhancing the therapeutic efficacy of these modalities, accompanied with the discussion on their biological effects and biosafety. The existing gap between fundamental research and clinical translation of these metal peroxide based nanotherapeutic technologies is finally discussed in depth.

## Graphical abstract

This progress report summarizes and discusses the most representative paradigms of metal peroxides in chemoreactive nanomedicine, particularly focusing on the underlying reaction mechanisms on enhancing therapeutic efficacy in versatile diseases and discussing biological effects and biosafety. The existing gap between fundamental research and clinical translation of these metal peroxide based nanotherapeutic technologies is finally discussed in depth.

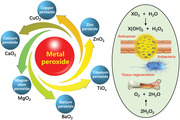

## 1 Introduction

The progress of material science has substantially contributed to clinical medicine by the development of versatile high‐performance therapeutic modalities for disease treatment.^[^ ^1^, ^2^, ^3^, ^4^, ^5^ ^]^ This interdisciplinary field promotes the construction of abundant biomaterials with intrinsically unique structure, composition, topology, physiochemical property, and biological effect for satisfying varied diagnostic, therapeutic, or theranostic clinical requirements.^[^ ^6^, ^7^, ^8^, ^9^, ^10^, ^11^ ^]^ Especially, nanosized biomaterials provide the bases for the march of nanobiotechnology and nanomedicine, which are generally in the formulas of organic, inorganic, or organic–inorganic hybrid nanosystems.^[^ ^12^, ^13^, ^14^ ^]^ Compared to the mostly explored organic nanosystems with high biocompatibility due to their comparable or similar composition to living creatures,^[^ ^15^, ^16^ ^]^ inorganic nanoparticles have attracted ever‐increasing attention because of their physiochemical property with specific response to external physical triggers, thus showing unique photonic, electric, acoustic, and magnetic properties.^[^ ^17^, ^18^, ^19^, ^20^ ^]^ Among these inorganic nanosystems, metal or metal oxides are a large category of biomaterials with numerous “star nanosystems” such as the well‐known Au nanoparticles with surface plasma resonance property and Fe_3_O_4_ nanoparticles with superparamagnetic property,^[^ ^21^, ^22^, ^23^ ^]^ which have entered either clinical‐trial stage on tumor therapy or clinical‐use phase for disease diagnosis, certificating high significance and prospects of metal‐involved nanosystems in biomedicine.

One of the targets of disease‐therapeutic development is to explore disease‐specific treatment modalities, which typically requires either high targeting/accumulation of therapeutic agents into lesion site or only exerting the therapeutic role in disease site rather than the healthy tissue. The rational design of targeting strategies has been developed for decades in which versatile targeting protocols have been proposed for enhancing the targeting efficiency.^[^ ^24^, ^25^ ^]^ However, the progress of such a targeting strategy is still far from satisfactory. For instance, the accumulation amounts of therapeutic nano‐agents into tumor tissue is still less than 10% because of the reticuloendothelial system,^[^ ^14^, ^26^, ^27^, ^28^ ^]^ which cannot avoid the severe side effects of toxic agents as accumulated into normal cells/tissues. The exploration of disease‐specific treatment by triggering in situ chemical reactions has aroused extensive research interest, especially in the scientific community of nanotechnology and nanomedicine.^[^ ^29^, ^30^, ^31^, ^32^ ^]^ Abundant nanoparticles that can trigger desirable chemical reactions for disease therapy are emerging in the forms of either nano‐catalysts or nano‐reactant. The catalytic medicine recently promotes the construction of several kinds of nano‐catalysts for chemoreactive nanotherapeutics, but very few nano‐reactants have been developed for disease treatment, which herein requires the invention of more reactive nano‐reactants that can induce chemical reactions in disease microenvironment for accomplishing disease‐specific chemoreactive nanotherapeutics.^[^ ^33^, ^34^, ^35^, ^36^ ^]^

**Figure 1:**
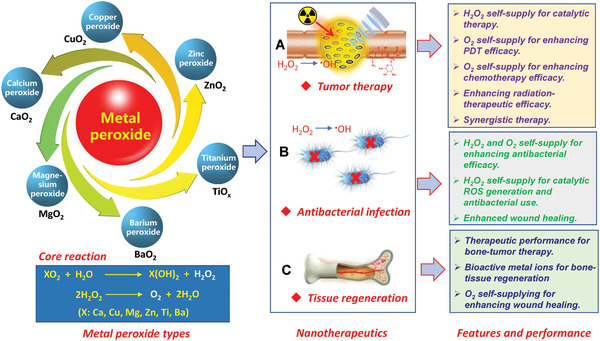
Schematic illustration of metal peroxide paradigms and underlying core reactions for chemoreactive nanotherapeutics with the specific feature and therapeutic performance in tumor therapy, antibacterial infection, and tissue regeneration.

Metal peroxides are typically composed of metal ions and peroxo groups, which can react with H_2_O to produce hydrogen peroxide (H_2_O_2_).^[^ ^37^, ^38^, ^39^, ^40^ ^]^ The post‐generated H_2_O_2_ is useful for numerous biomedical applications.^[^ ^41^, ^42^, ^43^ ^]^ For example, H_2_O_2_ can act as the reactants of Fenton‐like catalytic reaction in catalytic medicine for large production of highly toxic hydroxyl radicals (•OH).^[^ ^44^, ^45^ ^]^ In addition, H_2_O_2_ can self‐decompose to produce oxygen (O_2_) for enhancing the therapeutic efficacy of different O_2_‐involved modalities such as photodynamic therapy (PDT) and radiation therapy (RT).^[^ ^46^, ^47^, ^48^ ^]^ Therefore, metal peroxide can act as the solid precursor for generating O_2_ and H_2_O_2_. Importantly, the intrinsic metal‐ion component in metal peroxides participates in versatile biological procedures such as biological reaction or tissue‐regeneration process. On this ground, metal peroxide based nanoparticles as one of the important but generally ignored categories of inorganic nanosystems represent an emerging nanosystem with their intrinsic physiochemical properties, reactive features, and biological effects for satisfying different requirements of biomedical applications.^[^ ^49^ ^]^ Based on the fast development of metal peroxide based nanosystems in chemoreactive disease nanotherapeutics very recently, this progress report summarizes and discusses the progress of the construction of versatile metal peroxide nanoparticles for disease‐specific chemoreactive nanotherapeutics (**Figure** **1**). These metal peroxide nanosystems include copper peroxide (CuO_2_), calcium peroxide (CaO_2_), magnesium peroxide (MgO_2_), zinc peroxide (ZnO_2_), barium peroxide (BaO_2_), and titanium peroxide (TiO*_x_*). Based on their reactivity for H_2_O_2_ and O_2_ production and metal ion based bioactivity, they have been broadly explored in different biomedical frontiers, including catalytic nanotherapeutics, PDT, RT, antibacterial infection, tissue regeneration, and some synergistically therapeutic applications. Their proprietary biological effects and biosafety are also discussed. Finally, we conclude this progress report with the discussion in depth on the current challenges and future prospects of building the bridges on the gap between fundamental research and clinical translation of metal peroxide based nanotherapeutic technologies.

## 2 Metal Peroxide Nanoparticles for H_2_O_2_/O_2_ Self‐Supplying Catalytic Medicine and Chemotherapy

Fenton reaction based catalytic nanotherapeutics have emerged as a distinctive tumor‐therapeutic modality with high tumor specificity.^[^ ^50^, ^51^, ^52^, ^53^ ^]^ Typically, it employs Fenton agents for triggering disproportionated reaction on converting tumor‐overexpressed H_2_O_2_ into highly toxic •OH for oxidative therapy. However, the low intratumoral H_2_O_2_ level of around 100 µm substantially limits the therapeutic efficiency of such a new catalytic reaction based nanotherapeutic. The traditional use of glucose oxidase for catalyzing glucose into H_2_O_2_ involves the O_2_ participation, which might induce the severe hypoxia of tumor.^[^ ^54^, ^55^, ^56^ ^]^ On this ground, the H_2_O_2_‐generating capability of metal peroxides provides the possibility for the design of cascade Fenton nanoagents for catalytic nanotherapeutics.

**Figure 2:**
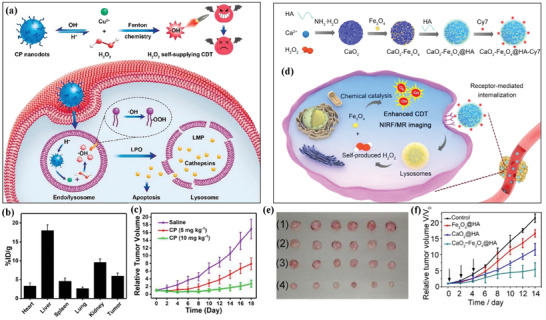
a) Schematic illustration of the detailed procedure regarding CuO_2_ nanoparticles for producing the specific effect of H_2_O_2_ self‐generation and Cu^2+^‐catalyzed Fenton reaction under the acidic tumor microenvironment. This procedure produced cytotoxic •OH to induce lysosomal lipid peroxidation and cause the apoptosis of cancer cells. b) Biodistribution assay of Cu component in organs and tumor after intravenous administration of CuO_2_ nanodots. c) Relative tumor‐volume change after the injection of CuO_2_ nanodots at two doses (5 and 10 mg kg^−1^) for prolonged durations. Reproduced with permission.^[^ ^57^ ^]^ Copyright 2019, American Chemical Society. d) The scheme of the fabrication procedure of CaO_2_‐Fe_3_O_4_@ hyaluronate acid (HA) nanoparticles, and the underlying therapeutic mechanism of H_2_O_2_ self‐supplying Fenton‐like catalytic reactions for producing •OH to induce cancer‐cell death with near‐infrared fluorescence (NIRF)/magnetic resonance imaging (MRI) dual‐imaging guidance and monitoring performance. e) Photographic images of excised tumors after varied treatment protocols, including 1) saline, 2) Fe_3_O_4_@HA nanoparticles, 3) CaO_2_@HA nanoparticles, and 4) CaO_2_‐Fe_3_O_4_@HA nanoparticles. f) Tumor‐volume changes after varied treatments as exhibited in the figure with prolonged durations. Reproduced with permission.^[^ ^58^ ^]^ Copyright 2020, American Chemical Society.

Multifunctional copper peroxide (CuO_2_, CP) nanodots were facilely synthesized in an aqueous reaction system containing CuCl_2_, H_2_O_2_, and sodium hydroxide (**Figure** **2**a).^[^ ^57^ ^]^ Poly(vinylpyrrolidone) (PVP) was involved in this reaction, which not only controlled the particle size of nanodots, but also provided the surface modification for guaranteeing the high stability of nanodots in physiological conditions. Their small particle size of around 5 nm enabled efficient tumor accumulation via the typical enhanced permeability and retention (EPR) effect (Figure 2b). The constructed CuO_2_ nanodots triggered chemical reaction to produce H_2_O_2_ by reaction with H_2_O, and the presence of Cu^2+^ as catalysts triggered Fenton‐like reaction to generate highly toxic •OH using self‐supplying H_2_O_2_ as the reactant (Figure 2a). The produced •OH radicals induced lysosomal membrane permeabilization‐mediated cancer‐cell apoptosis by lysosomal lipid peroxidation. The in vivo therapeutic evaluation of U87MG tumor xenograft exhibited high tumor‐suppression efficacy at a dose‐dependent manner (Figure 2c).^[^ ^57^ ^]^

Despite CuO_2_ nanoparticles themselves can act as both H_2_O_2_ supplier and catalytic center, the potential toxicity of Cu ions to normal cells/tissues at high dose might induce the critical issue of low biosafety.^[^ ^59^, ^60^, ^61^ ^]^ Comparatively, CaO_2_ nanoparticles are preferable because of the higher biocompatibility of Ca^2+^ ions that are abundantly present in vivo. However, the chemically inert Ca component in CaO_2_ nanoparticles cannot trigger chemical reaction, signifying that they should be integrated with other Fenton agents for achieving therapeutic purposes. On this ground, CaO_2_ nanoparticles were integrated with extensively explored and highly biocompatible Fe_3_O_4_ Fenton nano‐agents with the assistance of hyaluronate acid (HA) for the construction of CaO_2_‐Fe_3_O_4_@HA composite nanoparticles (Figure 2d), which achieved H_2_O_2_ self‐supplying and Fenton‐based tumor nanotherapeutics.^[^ ^58^ ^]^ HA was chosen based on its strong affinity to CaO_2_ and Fe_3_O_4_ by the coordination of carboxyl groups of HA with Ca^2+^ and Fe^3+^. The constructed CaO_2_‐Fe_3_O_4_@HA nanoparticles initially reacted with H_2_O to produce H_2_O_2_, and the Fe_3_O_4_ component further converted H_2_O_2_ into •OH for inducing cancer‐cell death. Based on in vivo 4T1 tumor‐bearing mice model, the intravenous administration of CaO_2_‐Fe_3_O_4_@HA nanoparticles achieved 69.08% tumor‐suppression rate (Figure 2e,f), much higher as compared to either Fe_3_O_4_@HA nanoparticles (19.44%) and CaO_2_@HA nanoparticles (29.39%).^[^ ^58^ ^]^ Similarly, transferrin‐modified MgO_2_ nanosheets were constructed for H_2_O_2_ self‐supplying and Fenton reaction based oxidative therapy.^[^ ^62^ ^]^ The initial reaction of MgO_2_ with H_2_O produced H_2_O_2_, which damaged the transferrin structure to release the trapped Fe^3+^. The release Fe^3+^ triggered Fenton reaction using pre‐generated H_2_O_2_ as the reactant to generate highly toxic •OH radicals, inducing cancer‐cell death in vitro and tumor inhibition in vivo.

**Figure 3:**
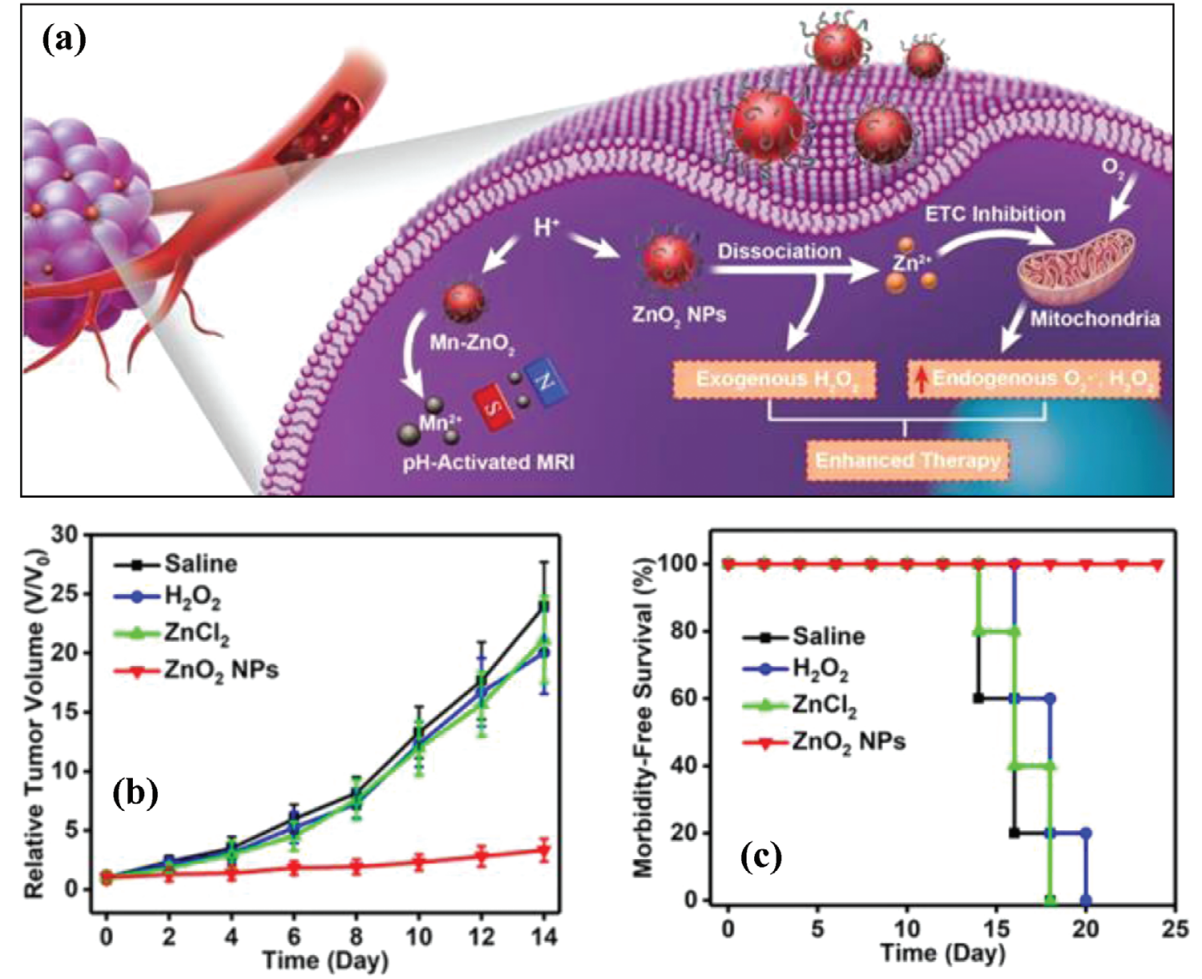
a) Schematic illustration of the underlying mechanism of ZnO_2_ nanoparticles for tumor therapy. ZnO_2_ nanoparticles reacted with H_2_O under mildly acidic condition to produce both H_2_O_2_ and Zn^2+^. The post‐produced H_2_O_2_ was toxic to cancer cells, and the released Zn^2+^ enhanced the O_2_^•−^ production in mitochondria via inhibiting the electron transport chain, further improving the oxidative stress based cancer nanotherapeutics. Mn ions were doped into ZnO_2_ nanoparticles for achieving pH‐responsive MR imaging based on paramagnetic property of Mn centers. b) Relative tumor‐volume change and c) survival curves of tumor‐bearing mice with prolonged time after the treatment with different agents as shown in the figures. Reproduced with permission.^[^ ^63^ ^]^ Copyright 2019, lvyspring international Publisher.

In addition, PVP‐modified ZnO_2_ (PVP‐ZnO_2_) nanoparticles were synthesized by direct reaction between Zn(OAc)_2_, PVP, and H_2_O_2_.^[^ ^63^ ^]^ The constructed PVP‐ZnO_2_ nanoparticles initially reacted with H_2_O to produce both H_2_O_2_ and Zn^2+^ under the mildly acidic tumor microenvironment. Especially, this reaction produced two independent effects for enhancing intracellular oxidative stress (**Figure** **3**a). On one hand, the produced toxic H_2_O_2_ acted as the exogenous reactive oxygen species (ROS) for inducing cancer‐cell death. On the other hand, the released Zn^2+^ promoted the production of endogenous ROS in mitochondria in the form of superoxide anion free radicals (O_2_^•−^), inducing the synergistic effect for enhancing oxidative stress based cancer‐cell killing. The in vivo therapeutic efficacy was demonstrated on U87MG tumor xenograft, which exhibited a high tumor‐suppressing effect (Figure 3b) after the administration of ZnO_2_ nanoparticles with the improved survival rate (Figure 3c). This work demonstrates the synergistic effect of augmenting both exogenous and endogenous ROS production on combating cancer based on ZnO_2_ nanoparticles.

**Figure 4:**
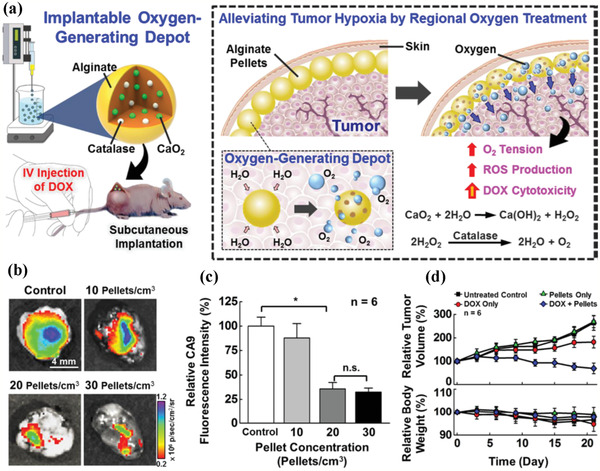
a) Schematic illustration of the fabricated alginate pellets with the encapsulated CaO_2_ nanoparticles and catalase, and the underlying mechanism for O_2_ generation‐enabled tumor‐hypoxia alleviation and DOX cytotoxicity enhancement. b) In vivo fluorescent imaging of hypoxia marker CA9 by HypoxiSense680 and c) corresponding fluorescence intensity in tumor after being treated with varied alginate pellets. d) Relative tumor‐volume and body‐weight changes after different treatments for prolonged durations. Reproduced with permission.^[^ ^67^ ^]^ Copyright 2016, American Chemical Society.

The tumor hypoxia has been demonstrated to lower the chemotherapeutic efficacy.^[^ ^64^, ^65^, ^66^ ^]^ In order to alleviate the tumor hypoxia and enhance the chemotherapeutic outcome of doxorubicin (DOX), an oxygen‐generating depot was constructed by directly encapsulating CaO_2_ nanoparticles and catalase into the matrix of alginate pellets (**Figure** **4**a).^[^ ^67^ ^]^ After the implantation of this multifunctional alginate pellet into the tissue close to tumor, the reaction of CaO_2_ and H_2_O initially produced H_2_O_2_. Because the decomposition rate of H_2_O_2_ into O_2_ was low, catalase was used to accelerate H_2_O_2_ decomposition and O_2_ generation. The O_2_ production alleviated tumor hypoxia and subsequently enhanced the efficacy of DOX chemotherapy. To visually show the degree of tumor‐hypoxia alleviation, the fluorescence‐imaging agent HypoxiSense 680 was used to characterize the hypoxia marker CA9. The in vivo fluorescent imaging and corresponding fluorescence intensity in tumor exhibited that the CA9 fluorescence intensity was substantially decreased after the implantation of oxygen‐generating depot (Figure 4b,c), demonstrating the desirable tumor hypoxia‐alleviating effect. Therefore, the chemotherapeutic efficacy of intravenously administrated DOX was strengthened as proven by the enhanced tumor‐suppression rate and less body‐weight loss (Figure 4d).^[^ ^67^ ^]^ This paradigm demonstrates the effectiveness of metal peroxide induced O_2_ production for boosting the chemotherapeutic outcome on combating cancer. In addition, the lipid‐coated CaO_2_/cisplatin nanomedicine was fabricated for modulating tumor microenvironment and strengthening cisplatin cytotoxicity against cancer cells.^[^ ^68^ ^]^ The CaO_2_ reaction with H_2_O induced O_2_ generation, pH elevation, and glutathione consumption, which further inactivated the O_2_‐dependent hypoxia‐inducible factor 1 (HIF‐1) to downregulate the multidrug resistance‐associate protein 2 (MRP2). Therefore, the anticancer efficacy of loaded cisplatin was significantly improved as demonstrated on a hepatocellular carcinoma xenograft model. In addition to the H_2_O_2_ production of CaO_2_ nanoparticles, their dissolution under mildly acidic condition could release Ca^2+^ intracellularly to induce calcium‐overloading stress in cancer cells and finally cause the cancer‐cell death.^[^ ^69^ ^]^

**Figure 5:**
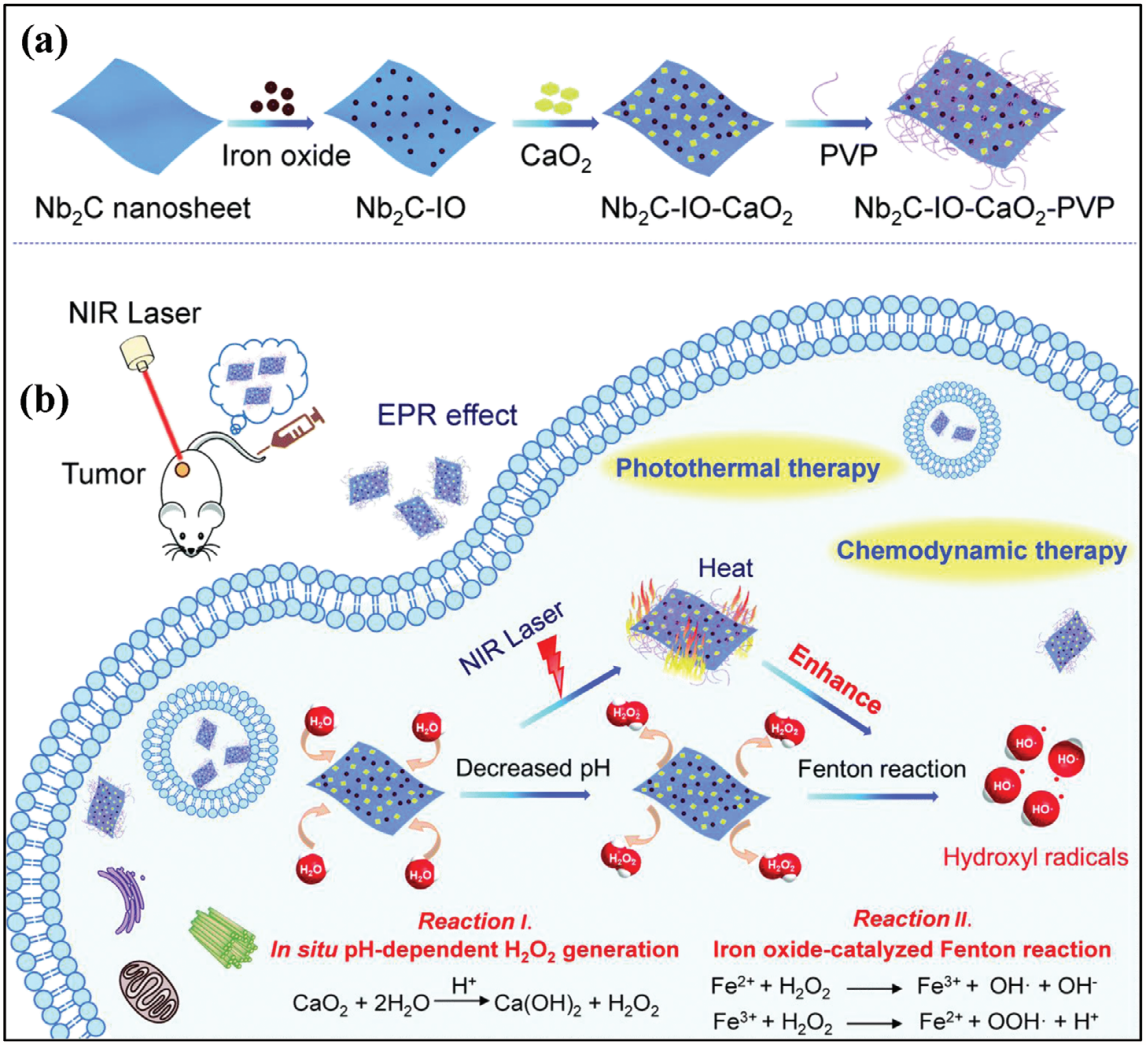
The scheme of a) loading CaO_2_ and Fe_3_O_4_ nanoparticles onto the surface of 2D Nb_2_C MXene for b) photothermal‐enhanced catalytic nanotherapeutics, including the detailed procedures of CaO_2_ reaction with H_2_O for H_2_O_2_ production, Fe_3_O_4_‐catalyzed Fenton reaction with pre‐produced H_2_O_2_ as the reactant, and NIR‐induced photothermal hyperthermia on synergistically enhancing ROS‐induced oxidative tumor nanotherapeutics Reproduced with permission.^[^ ^75^ ^]^ Copyright 2019, The Royal Society of Chemistry.

Metal peroxide nanoparticles enabled nanotherapeutics can be synergistically enhanced by versatile therapeutic modalities based on fast progress of theranostic nanomedicine regarding synergistic therapeutic modalities.^[^ ^70^, ^71^, ^72^, ^73^, ^74^ ^]^ For instance, metal oxide‐involved catalytic reactions are influenced by local temperature change, where the high temperature can accelerate the reaction rates and degrees, inducing improved production of therapeutic species. On this ground, we recently loaded CaO_2_ and Fe_3_O_4_ nanoparticles onto the large surface of 2D Nb_2_C MXene (**Figure** **5**).^[^ ^75^ ^]^ Similar to above‐mentioned discussion, the co‐presence of CaO_2_ and Fe_3_O_4_ nanoparticles triggered H_2_O_2_ self‐supplying Fenton reaction to produce •OH. Importantly, the 2D Nb_2_C MXene matrix has been extensively demonstrated as the high‐performance photothermal nanoagents,^[^ ^76^, ^77^, ^78^ ^]^ which responds to external near infrared (NIR) irradiation for converting photonic energy into thermal energy. Because Fenton reaction is temperature‐dependent, the NIR‐triggered photothermal conversion mediated by Nb_2_C MXene substantially enhanced CaO_2_/Fe_3_O_4_‐involved Fenton reaction degree/rate, achieving synergistic therapeutic outcome with a high tumor‐suppression rate as demonstrated in vivo on tumor xenograft.^[^ ^75^ ^]^

**Figure 6:**
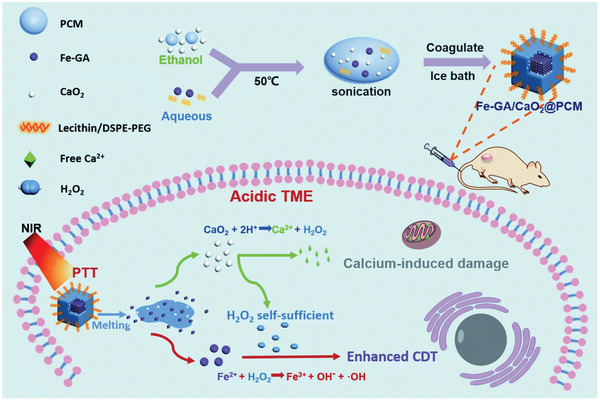
Schematic illustration on the construction of Fe‐GA/CaO_2_@PCM nanoparticles and the detailed mechanism of H_2_O_2_ self‐sufficient catalytic nanotherapeutics based on Fenton reaction with photothermal synergy Reproduced with permission.^[^ ^79^ ^]^ Copyright 2020, The Royal Society of Chemistry.

The loading of CaO_2_ nanoparticles and Fenton nanocatalysts onto the surface of 2D MXene could not avoid the pre‐reaction of CaO_2_ nanoparticles with H_2_O during blood circulation. This critical issue was partially solved by encapsulating CaO_2_ nanoparticles and iron‐gallic acid (Fe‐GA; Fenton nanoagent) into thermal‐responsive organic phase‐change materials (PCMs) with a melting point of 46 °C (**Figure** **6**).^[^ ^79^ ^]^ The PCM layer acted as the “gatekeeper” to firmly seal both CaO_2_ and Fe‐GA in the matrix when the surrounding temperature was lower than the melting point, which avoided the reaction of CaO_2_ and H_2_O. After entering the tumor tissue, the external NIR irradiation was converted into thermal energy by Fe‐GA to melt the PCMs, which exposed CaO_2_ to aqueous condition for producing H_2_O_2_. Fe‐GA as the Fenton agent further reacted with self‐sufficient H_2_O_2_ to produce •OH for killing cancer cells. Importantly, the photothermal effect played the specific role of triggering PCMs melting, accelerating Fenton reaction and further synergistically enhancing the Fenton reaction based nanotherapeutic efficacy, which was demonstrated by the in vivo mice‐bearing HeLa tumor model where the synergistic photothermal ablation and sequential catalytic reaction based nanotherapeutics achieved the highest tumor‐inhibiting outcome.^[^ ^79^ ^]^ The emerging of metal peroxide nanoparticles in biomedicine is still in the infancy, therefore the paradigms on their reactive nanotherapeutic‐based synergistic therapy are still very rare. Comparatively, nanomedicine‐based diverse therapeutic modalities (e.g., photothermal therapy, PDT, RT, sonodynamic therapy, chemotherapy, magnetic ablation) have been combined with various other therapeutic protocols for achieving higher therapeutic synergy.^[^ ^80^ ^]^ Therefore, it is expected that more metal peroxide based synergistic therapeutic modalities would be developed and explored in the following researches.

## 3 Metal Peroxide Nanoparticles for Enhanced Photodynamic and Radiation Therapy

It has been solidly demonstrated that the therapeutic outcome of PDT strongly depends on the O_2_ level in tumor,^[^ ^81^, ^82^, ^83^ ^]^ but the tumor hypoxia limits the PDT efficacy. The O_2_‐consuming PDT procedure can worsen the hypoxia degree, possibly causing the tumor metastasis and resistance to many therapeutic modalities such as PDT, RT, chemotherapy, and catalytic medicine.^[^ ^84^, ^85^ ^]^ Versatile nanotechnology‐enabled O_2_‐supplying strategies have been developed for alleviating tumor hypoxia, among which the direct conversion of tumor‐overexpressed H_2_O_2_ into O_2_ has been mostly explored, including the typical use of catalase and Mn‐based nanocatalysts.^[^ ^86^, ^87^, ^88^, ^89^ ^]^ The intrinsically low H_2_O_2_ level in tumor, however, severely limits the O_2_‐production efficacy.

**Figure 7:**
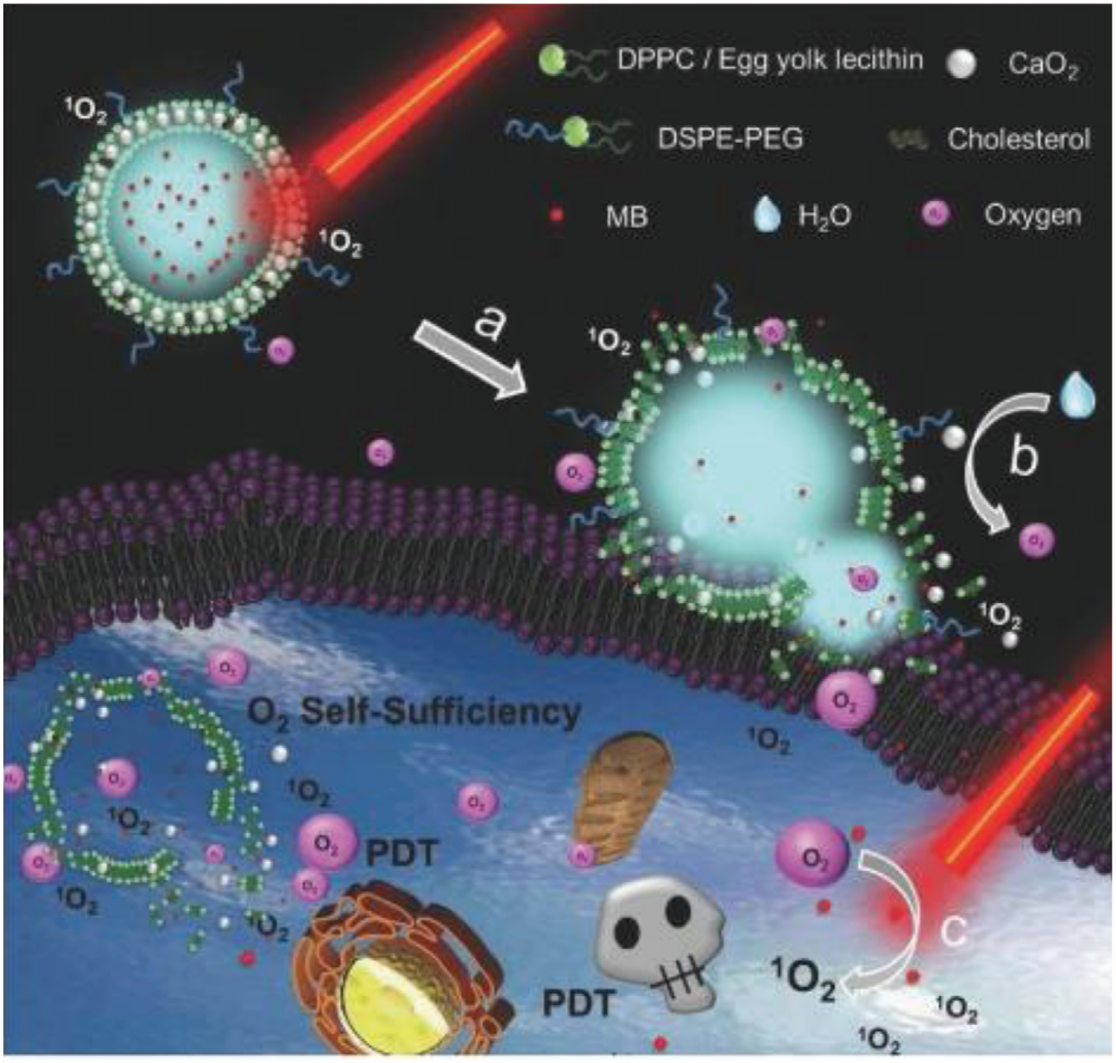
Schematic illustration of the detailed composition of LipoMB/CaO_2_ nanoparticles and their specific functionality for O_2_ self‐supplying photodynamic tumor therapy. The therapeutic procedure includes the initial light‐triggered MB‐based PDT for producing ^1^O_2_, which induced the oxidation of phospholipid bilayer to further enhance O_2_ production by enabling the direct reaction of CaO_2_ and H_2_O. The large O_2_ production strengthened the second‐stage light‐triggered PDT efficacy because of the alleviated tumor hypoxia. Reproduced with permission.^[^ ^90^ ^]^ Copyright 2017, John Wiley and Sons.

CaO_2_ nanoparticles can initially react with H_2_O to produce Ca(OH)_2_ and H_2_O_2_. The post‐generated H_2_O_2_ can then self‐decompose to generate O_2_ and H_2_O, by which CaO_2_ nanoparticles act as the O_2_ solid precursor. Therefore, the rational integration of CaO_2_ and photosensitizers can achieve O_2_ self‐supplying and strengthen photodynamic tumor therapy. On this ground, CaO_2_ nanoparticles and methylene blue (MB) photosensitizers were co‐loaded into a liposome to construct a nanosized photosensitizer (designated as LipoMB/CaO_2_).^[^ ^90^ ^]^ After entering the tumor tissue, the first‐stage light irradiation produced ^1^O_2_ to oxidize the phospholipid bilayer and induce the liposome break, enabling the direct interaction of CaO_2_ and H_2_O to produce H_2_O_2_. Accelerated O_2_ production was achieved by self‐decomposition of post‐generated H_2_O_2_, which substantially alleviated the tumor hypoxia. This effect further enhanced the PDT efficacy after the second‐stage light irradiation on MB photosensitizers (**Figure** **7**). Based on the in vivo 4T1 tumor animal model, the high tumor‐suppression efficacy was achieved by the administration of LipoMB/CaO_2_ nanoparticles followed by dual‐stage light irradiation.^[^ ^90^ ^]^ To avoid the pre‐reaction of CaO_2_ nanoparticles and H_2_O during the circulation within the blood vessel, the surface of CaO_2_ nanoparticles was coated with a pH‐sensitive methacrylate‐based co‐polymer with a tertiary amine residue that was stable at pH of higher than 7.4 but unstable at lower pH values.^[^ ^91^ ^]^ The dissolution of surface polymer under acidic condition triggered the reaction of CaO_2_ and H_2_O to produce O_2_. This effect substantially enhanced the tumor pO_2_ level of 6.5 mm Hg after the administration of CaO_2_ for 10–30 min, resulting in the improved PDT efficacy against in vivo human xenograft MIA‐PaCa‐2 pancreatic tumors. In addition to O_2_ generation enhanced PDT efficacy, the released Ca^2+^ was demonstrated to induce calcium overload in mitochondrial, and the O_2_‐induced tumor‐hypoxia alleviation decreased the tumor metastasis.^[^ ^92^ ^]^ Similarly, multifunctional CaO_2_/MnO_2_@PDA‐MB (PDA: polydopamine; MB: methylene blue) nanosheet was constructed for self‐production of O_2_ to mitigate tumor hypoxia and enhance PDT efficacy against tumor.^[^ ^93^ ^]^ In addition, the CaO_2_‐induced H_2_O_2_ and O_2_ self‐applying approach was employed for augmenting the therapeutic efficacy of both CDT (using H_2_O_2_ as the reactant) and PDT ⌈using O_2_ as the singlet oxygen ( ^1^O_2_) source⌉ by constructing manganese silicate‐supported CaO_2_ and indocyanine green (ICG) in phase‐changeable material lauric acid.^[^ ^94^ ^]^

**Figure 8:**
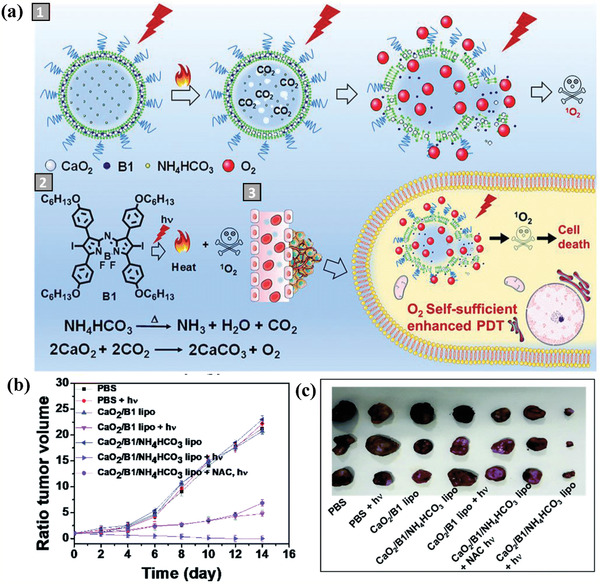
a) Schematic illustration of CaO_2_/B1/NH_4_HCO_3_ liposomes for O_2_ self‐supplying photodynamic tumor therapy, including 1) detailed O_2_ generation procedure from liposome, 2) the underlying chemical procedure of O_2_ production, and 3) intracellular O_2_ self‐production‐enhanced PDT for inducing cancer‐cell death. b) Tumor‐volume changes of tumor‐bearing mice after different treatments as indicated in the figure, and c) corresponding photographic tumor images at the end of varied treatments. Reproduced with permission.^[^ ^95^ ^]^ Copyright 2019, The Royal Society of Chemistry.

Different from the direction reaction between CaO_2_ and H_2_O to produce H_2_O_2_ with the following H_2_O_2_ decomposition to generate O_2_, CaO_2_, and NH_4_HCO_3_ were co‐loaded into a liposome for a different reaction pathway on O_2_ production.^[^ ^95^ ^]^ Especially, the photosensitizer B1 (hydrophobic halogenated aza‐BODIPY) was also encapsulated into the liposome for photonic oxidative therapy. The constructed CaO_2_/B1/NH_4_HCO_3_ liposomes were initially triggered by light irradiation for producing B1‐enabled photothermal effect to decompose NH_4_HCO_3_ component, which produced CO_2_ to be further reacted with CaO_2_ nanoparticles for rapidly generating O_2_ (**Figure** **8**a). This strategy can overcome the drawback of low O_2_‐generating rate from the decomposition of H_2_O_2_ as originated from the reaction between CaO_2_ and H_2_O. Therefore, the similar O_2_ generation induced tumor‐hypoxia alleviation was achieved for further enhancing the PDT of tumor by activating the loaded B1 photosensitizers, which was demonstrated in vivo on HeLa tumor‐bearing nude mice where the CaO_2_/B1/NH_4_HCO_3_ liposomes treated group with light irradiation achieved the highest tumor‐inhibiting outcome (Figure 8b,c).^[^ ^95^ ^]^ This paradigm provides an alternative strategy for the design of some specific chemical reactions of metal peroxides for satisfying different biomedical application requirements.

In addition to the specific functionality for improving the PDT efficacy by O_2_ self‐supplying, metal peroxide nanoparticles are also effective for enhancing the efficacy of RT. For instance, polyacrylic acid (PAA)‐modified titanium peroxide (TiO*_x_*) nanoparticles (designated as PAA‐TiO*_x_* NPs) expedited the ROS production after exposure to X‐ray irradiation, which exhibited substantially enhanced pancreatic tumor‐growth inhibition as compared to PAA‐TiO*_x_* nanoparticles alone treatment or single X‐ray irradiation.^[^ ^96^ ^]^ Under X‐ray irradiation, the Ba^2+^ in the lattice of chelator‐modified barium peroxide (BaO_2_) nanoparticles was sensitized to directly covert peroxide groups into cytotoxicity •OH radicals by emitting electrons, which induced DNA damage of cancer cells.^[^ ^97^ ^]^ The produced ROS by X‐ray radiation also triggered Ba^2+^ release by destroying the chemical structure of chelators, which further inhibited the potassium channel to suppress the cancer‐cell proliferation.

It has been well demonstrated that the tumor hypoxia significantly inhibits the efficacy of RT. The development of versatile nanotechnology‐enabled oxygenation and tumor‐hypoxia alleviation has been proven to be effective in strengthening tumor radiation therapy.^[^ ^98^, ^99^, ^100^, ^101^ ^]^ Considering that metal peroxides can generate oxygen as demonstrated to improve the O_2_‐dependent PDT efficacy, it is highly expected that these metal peroxide nanoparticles would be developed for enhancing radiation‐based therapeutic efficacy by in situ O_2_ production and tumor‐hypoxia alleviation.

## 4 Metal Peroxide Nanoparticle for Antibacterial Nanotherapeutics

Bacterial infection is one of the critical clinical issues threatening the health of human beings.^[^ ^102^, ^103^ ^]^ It has been demonstrated that ROS is effective in treating bacterial infections by inducing oxidative stress.^[^ ^104^, ^105^, ^106^ ^]^ Now that the above‐mentioned metal peroxides can produce ROS by either catalytic nanotherapeutics or PDT on combating cancer, it is highly expected that the similar strategy on ROS generation would be further extended to treat bacterial infection. On this ground, CaO_2_ nanocrystals and corresponding aggregates with uniform morphology and adjustable size were synthesized by a simple we‐chemical procedure.^[^ ^107^ ^]^ By using PVP as the stabilizer, CaO_2_ spherical aggregates with the size range of 15–100 nm were fabricated for evaluating their anti‐anaerobic bacterial activity. Especially, these CaO_2_ aggregates exhibited size‐dependent antibacterial effect because the H_2_O_2_ and O_2_ production was also size‐dependent.

**Figure 9:**
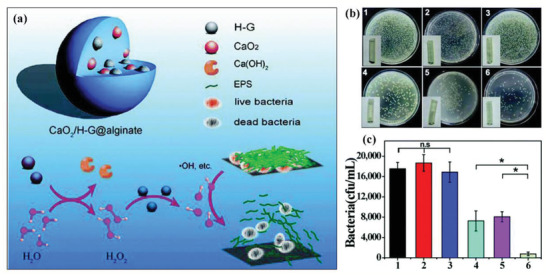
a) Schematic illustration on the detailed components of the constructed CaO_2_/H‐G@alginate depots and the underlying mechanism on treating bacterial infection. b) The bacteria as separated from implanted area on the mice with the inset images showing the corresponding catheters. c) The survival bacteria number of the wound tissue in different treatment groups. The numbers 1–6 in (b) and (c) respectively represent the groups of blank, alginate, H‐G@alginate, CaO_2_@alginate, mixed depots, and CaO_2_/H‐G@alginate. Reproduced with permission.^[^ ^108^ ^]^ Copyright 2018, The Royal Society of Chemistry.

In addition, CaO_2_ and hemin‐loading graphene (G‐H) were integrated into an alginate (designated as CaO_2_/H‐G@alginate) for bacterial infection treatment (**Figure** **9**a).^[^ ^108^ ^]^ The antibacterial procedures includes the reaction of CaO_2_ and H_2_O to produce Ca(OH)_2_ and H_2_O_2_, conversion of H_2_O_2_ into ROS by the encapsulated H‐G, and ROS‐induced biofilm damages. In vivo animal model of implant‐related periprosthetic infection was established with the following subcutaneous implantation of CaO_2_/H‐G@alginate. The skin wounds in CaO_2_/H‐G@alginate treatment group exhibited the fastest healing rate. In addition, the contaminated medical catheters as taken out in CaO_2_/H‐G@alginate treatment group exhibited the substantially damaged biofilm. More than 90% bacteria were efficiently killed after CaO_2_/H‐G@alginate treatment (Figure 9b,c), much higher than other treatment groups. This work provides the new biomedical applications of metal peroxide nanoparticles in antibacterial use by rational design of metal peroxide reaction, H_2_O_2_ production, and adequate H_2_O_2_ use. O_2_‐generating polycaprolactone (PCL) antimicrobial nanofibers were fabricated by CaO_2_ integration, which exhibited short‐time inhibitory performance on the proliferation of *Escherichia coli* and *Staphylococcus epidermidis* and kept relatively long‐time tissue‐integration behavior.^[^ ^109^ ^]^

## 5 Metal Peroxide Nanoparticles for Tissue Regeneration

**Figure 10:**
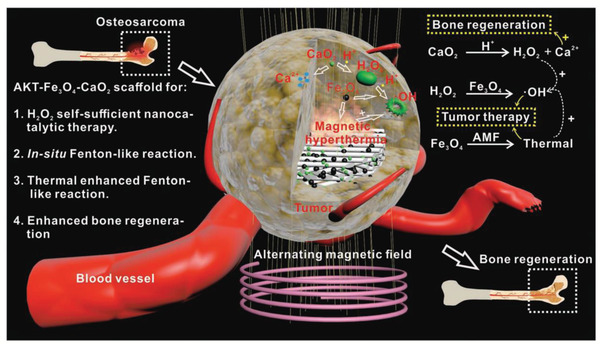
The specific functionality of CaO_2_ nanoparticles in bone‐tissue regeneration. The AKT‐Fe_3_O_4_‐CaO_2_ theragenerative biomaterial scaffold was initially constructed by directly loading Fe_3_O_4_ and CaO_2_ nanoparticles into the 3D‐printing scaffolds. CaO_2_ nanoparticles reacted with H_2_O to produce H_2_O_2_, which acted as the reactant for further Fe_3_O_4_‐catalyzed Fenton reaction under the mildly acidic environment of bone tumor. The external alternating magnetic field activated magnetic Fe_3_O_4_ nanoparticles for locally elevating the tumor temperature, which enhanced the Fenton reaction‐induced ROS production efficacy because such a Fenton reaction is temperature‐dependent. Enhanced bone regeneration was achieved by the CaO_2_ component because it could provide Ca^2+^ for participating in the bone‐regenerating process. Reproduced with permission.^[^ ^121^ ^]^ Copyright 2019, John Wiley and Sons.

Metal ions are featured with their intrinsic bioactivity for satisfying different biomedical application requirements.^[^ ^110^, ^111^, ^112^, ^113^, ^114^, ^115^, ^116^, ^117^, ^118^ ^]^ For instance, Ca^2+^ ions are the important component of bone, signifying that CaO_2_ nanoparticles might be applicable for tissue engineering.^[^ ^119^, ^120^ ^]^ Based on the consideration that CaO_2_ nanoparticles are typically designed for tumor‐therapeutic purposes, we recently loaded CaO_2_ nanoparticles into the matrix of 3D printing akermanite scaffold with the simultaneously integrated magnetic Fe_3_O_4_ nanoparticles (designated as AKT‐Fe_3_O_4_‐CaO_2_), which exerted the specific functionality for osteosarcoma treatment (**Figure** **10**).^[^ ^121^ ^]^ On the one hand, the fabricated theragenerative biomaterial AKT‐Fe_3_O_4_‐CaO_2_ efficiently killed bone‐tumor cells by magnetic hyperthermia enhanced sequential catalytic reaction. Like the aforementioned discussion on the combination of Fe_3_O_4_ and CaO_2_ for H_2_O_2_ self‐supplying Fenton reaction based ROS production, the external alternative magnetic field activated magnetic Fe_3_O_4_ to generate thermal effect for further enhancing the ROS‐production efficacy, resulting in the substantial bone tumor‐cell death as demonstrated both in vitro and in vivo. On the other hand, the integrated CaO_2_ nanoparticles as Ca^2+^ ion pools released Ca^2+^ for inducing the strengthened bone regeneration on repairing bone defects. This work demonstrates the function of Ca component in metal peroxide for bone‐tissue regeneration, accompanying with the specific therapeutic performance of metal peroxide.

**Figure 11:**
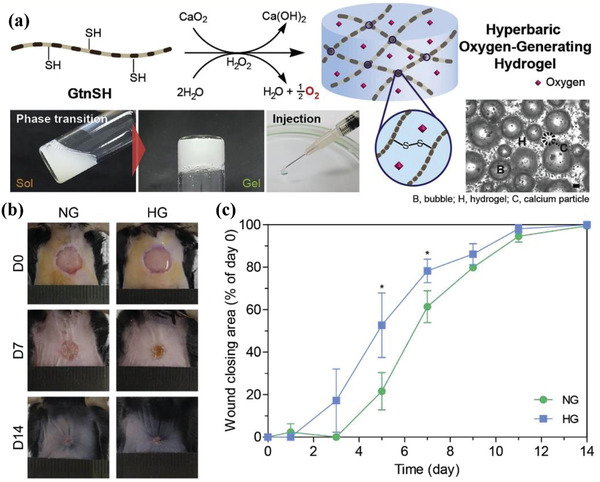
a) The scheme of HOG hydrogel synthesis and gel formation, and the photographic images showing the sol–gel phase transformation, facile hydrogel injection, and generated oxygen bubbles within the hydrogel matrix. b) Digital photos of wounds and c) corresponding quantitative wound closure curves after the treatment with NG and HG (NG: normoxic gel, HG: hyperbaric gel). Reproduced with permission.^[^ ^128^ ^]^ Copyright 2018, Elsevier.

In addition to the H_2_O_2_/metal ions production by metal peroxide nanoparticles for bone‐tumor therapy and bone‐tissue regeneration, their O_2_ production capability can also be employed for tissue regeneration because O_2_ is a signaling molecule participating in cellular activity regulation and metabolism control such as the proliferation, migration, and differentiation of cells.^[^ ^122^, ^123^ ^]^ Especially, the O_2_ level elevation promotes the wound healing and tissue regeneration by influencing varied biological factors such as collagen synthesis and angiogenesis.^[^ ^124^, ^125^, ^126^, ^127^ ^]^ On this ground, CaO_2_ nanoparticles were integrated into a thiolated gelation (GtnSH)‐based hydrogel for producing a specific hyperbaric oxygen‐generating (HOG) hydrogel.^[^ ^128^ ^]^ The CaO_2_‐enabled oxidative cross‐linking chemical reaction generated disulfide bonds to accelerate the hydrogel network formation (**Figure** **11**a) during the decomposition procedure into H_2_O_2_ and O_2_ after reaction with H_2_O. The fabricated HOG hydrogels quickly elevated the O_2_ level to even hyperoxic levels with long sustaining period, such as 12 days in vitro and 4 h in vivo. Especially, the HOG hydrogels enhanced the in vitro proliferation bioactivity of HDFs (human dermal fibroblasts) and HUVECs (human umbilical vein endothelial cells), and accelerated the wound‐healing rate with substantially enhanced tissue infiltration and neovascularization as compared to the repairing performance of normoxic gel (Figure 11b,c). Therefore, such a CaO_2_‐functionalized HOG hydrogel features the prospects for tissue regeneration regarding wound healing and vascular disorders. By physically dispersing CaO_2_ into the biodegradable crosslinking cyanoacrylate, the O_2_‐generating property of CaO_2_ improved the dermal wound healing in vivo on a rat model.^[^ ^129^ ^]^ Similarly, sodium percarbonate and CaO_2_ were used for constructing O_2_‐generating wound dressings, which also exhibited the enhanced in vivo wound‐healing effect within eight weeks.^[^ ^130^ ^]^

For scaffold implantation, the low O_2_ level might induce cell necrosis and bacterial infection.^[^ ^109^, ^131^ ^]^ To solve this critical issue, calcium peroxide (CaO_2_) as the oxygen self‐sufficient and antimicrobial component, was coated on the bioceramic scaffolds, which exerted the controllable O_2_‐releasing behavior by varying the CaO_2_‐loading amount.^[^ ^132^ ^]^ The loaded CaO_2_ exhibited antibacterial bioactivity against *E. coli* and *Staphylococcus aureus*. In addition, the CaO_2_ addition induced higher alkaline phosphatase activity of Saos‐2 cells and improved apatite formation in simulated body fluid test. The endowed antibacterial performance and improved alkaline phosphatase bioactivity by CaO_2_ integration with O_2_ self‐sufficient property demonstrated the high potential of CaO_2_‐functionalized bioceramic scaffolds for bone‐tissue regeneration.^[^ ^132^, ^133^ ^]^ Especially, the CaO_2_‐mediated oxygen supply was used for the creation of amine‐rich substrates for 3D cell spheroid formation, representing a surface‐modification strategy of biomaterials.^[^ ^134^ ^]^ In addition, CaO_2_ laden gelatin methacryloyl hydrogel provided sufficient O_2_ to alleviate the metabolic stress of cardiac side population cells, promising their biomedical use in the regeneration of infarcted myocardial tissue.^[^ ^135^ ^]^

## 6 Biological Effects and Biosafety of Metal Peroxide Nanoparticles

The biological effects and biosafety of metal peroxide nanoparticles plays the determining role for their further clinical translation. Despite these metal peroxide nanoparticles have shown promising therapeutic performance in chemoreactive nanotherapeutics, the reactivity of these nanosystems and corresponding metal composition might induce some potential toxicity issues and side effects. On one hand, the high reactivity of metal peroxides could potentially react with surrounding H_2_O when circulating in the blood vessel. H_2_O_2_ is produced by such a chemical reaction, which might induce the toxicity to healthy cells/tissues because of the H_2_O_2_ toxicity.^[^ ^136^, ^137^ ^]^ In addition, the formation of metal oxides and sustained release of metal ions might also induce the potential toxicity because some metal‐ion species are toxic,^[^ ^59^, ^60^, ^61^ ^]^ such as Cu^2+^, Zn^2+^, and Ba^2+^, which could also damage the accumulated healthy tissues.^[^ ^138^, ^139^, ^140^, ^141^ ^]^

To solve the potential toxicity issue and side effects, two potential strategies are herein proposed for guiding the further fundamental research. For the undesirable reactivity, the adequate surface modification and nanocarrier encapsulation are suggested to control the reactivity of these metal peroxides, which is expected to only trigger the chemical reactions within the disease microenvironment rather than the blood vessels or healthy tissues. On the other hand, for the potential release of toxic metal ions, the controllable decomposition of metal peroxide nanoparticles should be achieved. For instance, the decomposition of metal peroxide only occurs in mildly acidic tumor condition rather than the normal neutral tissues. Of course the widely‐accepted targeting design should be fully considered and carefully designed because the possibly high accumulation of metal peroxide nanoparticles into lesion sites can mostly mitigate their influence and side effects to healthy cells and tissues.

The biocompatibility and biosafety of metal peroxide nanoparticles have been preliminarily explored in several paradigms. The related data are encouraging,^[^ ^57^, ^62^, ^75^, ^121^ ^]^ but they are still far away from the biosafety demonstration for guaranteeing further clinical translation. More systematic in vitro and in vivo biosafety evaluations should be conducted to provide solid data and evidences on biocompatibility and biosafety. The in vivo biodistribution, excretion, histocompatibility and hemocompatibility, and especially long‐term biological effects are expected to be revealed under the further systematic fundamental researches.

## 7 Conclusions and Outlook

**Figure 12:**
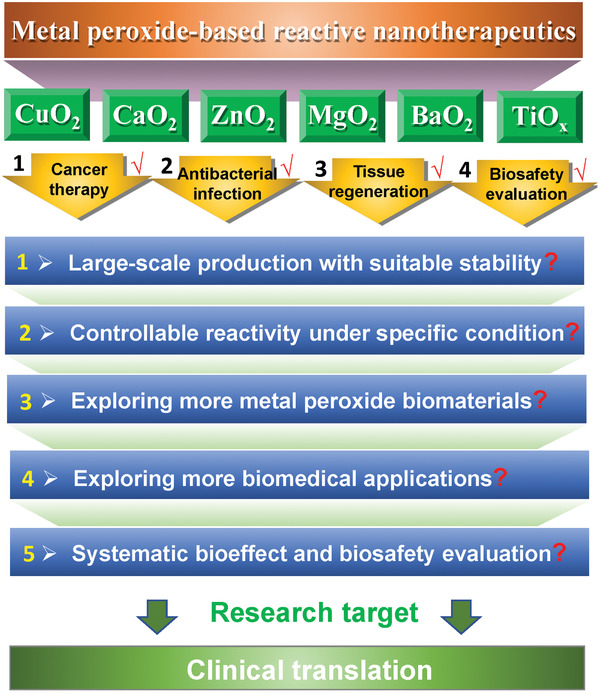
The conclusive scheme of the currently developed metal peroxide family members, their explored biomedical applications, and future development directions.

Significantly different from traditional metal oxides, the recently developed metal peroxide nanoparticles have attracted particular research interests in biomedicine because of their chemical reactivity, corresponding reaction products (e.g., H_2_O_2_ and O_2_), and specific biological effect of released metal ions. Versatile metal peroxide nanoparticles have been constructed for nanotherapeutics at current stage, such as CuO_2_, CaO_2_, MgO_2_, ZnO_2_, BaO_2_, and TiO*_x_*. They have been extensively explored in cancer therapy, antibacterial infection, and tissue regeneration (**Table** **1**). The high nanotherapeutic performance prospects their further clinical translation, provided that the following critical issues are adequately solved (**Figure** **12**).

**Table 1:** Selected paradigms of metal peroxide based biomaterials for versatile biomedical applications

No.	Metal peroxide	Reactive components	Biomedical application	Performance	Ref.
1	CuO_2_	Cu^2+^ and H_2_O_2_	Anticancer	H_2_O_2_ and Cu^2+^ self‐supplying for triggering effective Fenton reaction based tumor therapy	^[^ ^57^ ^]^
2	CaO_2_	Fe_3_O_4_ and H_2_O_2_	Anticancer	H_2_O_2_ self‐supplying for Fenton reaction based catalytic tumor eradication using Fe_3_O_4_ as Fenton nanocatalysts	^[^ ^58^ ^]^
3	ZnO_2_	Zn^2+^ and H_2_O_2_	Anticancer	The released Zn^2+^ promoted the production of endogenous ROS in mitochondria in the form of O_2_^•−^, inducing the synergistic effect for enhancing H_2_O_2_‐based cancer‐cell killing	^[^ ^63^ ^]^
4	MgO_2_	Fe^3+^ and H_2_O_2_	Anticancer	H_2_O_2_ self‐supplying for Fe^3+^‐triggered Fenton reaction and further oxidative stress based nanocatalytic tumor therapy	^[^ ^62^ ^]^
5	BaO_2_	Ba^2+^ and peroxide	Anticancer	X‐ray irradiation‐induce hydroxyl radical generation and Ba^2+^ for inhibiting the potassium channel to suppress the cancer‐cell proliferation	^[^ ^97^ ^]^
6	TiO*_x_*	TiO*_x_*	Anticancer	Enhancing the ROS production after exposure to X‐ray irradiation for substantially improved pancreatic tumor‐growth inhibition	^[^ ^96^ ^]^
7	CaO_2_	H_2_O_2_ and O_2_	Anticancer	Large O_2_ production for alleviating tumor hypoxia and subsequently enhancing the efficacy of DOX chemotherapy on combating tumor	^[^ ^67^ ^]^
8	CaO_2_	O_2_ and MB	Anticancer	O_2_ self‐supplying effect for enhancing the photodynamic tumor‐therapeutic efficacy	^[^ ^90^ ^]^
9	CaO_2_	CaO_2_, NH_4_HCO_3_, and O_2_	Anticancer	The reaction between CaO_2_ and NH_4_HCO_3_ (CO_2_ production) for O_2_ production and subsequently enhancing the PDT outcome	^[^ ^95^ ^]^
10	CaO_2_	H_2_O_2_ and hemin	Antibacteria	H_2_O_2_ self‐supplying Fenton reaction for ROS production for subsequent antibacterial infection, and accelerated wound healing	^[^ ^108^ ^]^
11	CaO_2_	O_2_ and H_2_O_2_	Antibacteria	O_2_‐generating PCL antimicrobial nanofibers with CaO_2_ for achieving inhibitory performance on the proliferation of *E. coli* and *S. epidermidis*	^[^ ^109^ ^]^
12	CaO_2_	Ca^2+^ and H_2_O_2_	Tissue regeneration	H_2_O_2_ self‐supplying Fenton reaction for killing bone‐tumor cells and Ca^2+^‐accelerated bone‐tissue regeneration	^[^ ^121^ ^]^
13	CaO_2_	O_2_	Tissue regeneration	O_2_‐generating HOG hydrogels for enhancing the in vitro proliferation bioactivity of HDFs and HUVECs and accelerating the wound‐healing rate	^[^ ^128^ ^]^
14	CaO_2_	H_2_O_2_, Fe_3_O_4_, and Nb_2_C	Anticancer	Synergistic 2D Nb_2_C MXene‐enabled photonic hyperthermia and H_2_O_2_ self‐supplying Fenton reaction based oxidative therapy on combating cancer	^[^ ^75^ ^]^
15	CaO_2_	H_2_O_2_ and Fe‐GA	Anticancer	Synergistic H_2_O_2_ production enabled efficient Fenton reaction based catalytic therapy and Fe‐GA‐enabled photothermal therapy on cancer treatment	^[^ ^79^ ^]^

For metal‐peroxide nanoparticle fabrication and storage, two challenges should be considered. Because of the high reactivity of metal peroxides, their well‐defined fabrication is highly difficult, resulting in the irregular morphology, uncontrollable particle size, and easy aggregation. The lab‐based production is currently difficult, not to mention the further large‐scale production and industrial translation. In addition, the easy reaction of metal peroxide nanoparticles with H_2_O makes their difficulty in storage because they can slowly react with surrounding water molecules to result in the uncontrollable nanoparticle quality for further biomedical use. It is expected that the advances of synthetic material chemistry would provide the adequate fabrication methodologies for controllable construction of metal peroxide nanoparticles with desirable key structural/compositional parameters, and develop desirable strategies for enhancing their stability for facile storage.

The reactivity of metal peroxide nanoparticles is still difficult to control at current stage, which means that their reaction can be easily triggered in the living creatures with aqueous environment. Therefore, the reaction productions are unavoidably present in the healthy tissues, causing the potential side effects. It is highly expected that the reactions should only be initiated just under the lesion condition. Therefore, some stimuli‐responsive strategies are rationally designed for achieving either endogenously (e.g., features of tumor microenvironment) or exogenously (e.g., photonic irradiation, acoustic exposure, radiation focusing) triggered reactions of metal peroxide nanoparticles, which strongly depends on the advances of nanosynthetic chemistry, nanobiotechnology, and nanomedicine.

The currently explored metal peroxide nanoparticles mainly include CuO_2_, CaO_2_, MgO_2_, ZnO_2_, BaO_2_, and TiO*_x_*, which exhibit different therapeutic performance and biological effect because of their varied reactivity and metal‐ion components. It is highly expected that more metal peroxide nanoparticles will be explored to satisfy different requirements of biomedical applications. More peroxide family members, in addition to inorganic metal peroxides, such as organic peroxide nanoparticles are expected to be synthesized with improved biocompatibility and comparable reactive performance. Based on the desirable reaction production participating in abundant disease evolutions (H_2_O_2_ and O_2_) and specific bioeffects of different metal ions, more biomedical applications will be explored in the following researches, in addition to the currently explored PDT, RT, chemotherapy, antibacterial infection, catalytic medicine, and tissue engineering.

As one of the most representative nanosystems with high reactivity and desirable reaction products, metal peroxide nanoparticles provide the unique bases for the rational design of versatile new therapeutic modalities on combating varied diseases. It is noted that the development of metal peroxide nanoparticles in biomedicine is only at the preliminary stage, but their specific and unique physiochemical properties for in situ reaction‐based nanotherapeutics, biological effects of reaction products/metal ions, and high therapeutic performance in disease treatment prospect their further progress in benefiting personalized and precise medicine.

## Conflict of Interest

The authors declare no conflict of interest.
